# The antecedents of biliary cancer: a primary care case–control study in the United Kingdom

**DOI:** 10.1038/sj.bjc.6604765

**Published:** 2008-11-18

**Authors:** M J Grainge, J West, M Solaymani-Dodaran, G P Aithal, T R Card

**Affiliations:** 1 Division of Epidemiology and Public Health, School of Community Health Sciences, University of Nottingham, Nottingham NG7 2UH, UK; 2 Nottingham Digestive Diseases Centre, Biomedical Research Unit, Nottingham NG7 2UH, UK; 3 Shahid Beheshti University of Medical Sciences, Research Institute for Endocrine Sciences, Tehran, Iran

**Keywords:** cholangiocarcinoma, gall bladder cancer, risk factors, non-steroidal anti-inflammatory drugs, database study

## Abstract

In a case–control study using a large UK primary care database, we found that non-steroidal anti-inflammatory drugs had no protective effect against biliary carcinomas (cholangiocarcinoma and gall bladder cancer). Increased risks were observed for cigarette smoking, diabetes, gallstone disease and obesity.

Cholangiocarcinoma has a 5-year survival rate of only 20% (Nathan *et al*, 2007). It is becoming more common in both the UK and the USA. At the same time, the incidence of the other cancer of the biliary epithelium (gall bladder cancer) has declined (West *et al*, 2006). Risk factors for cholangiocarcinoma include primary sclerosing cholangitis, gallstone disease (Ekbom *et al*, 1993), HCV infection (Donato *et al*, 2001), diabetes and obesity (Welzel *et al*, 2007). Gall bladder cancer has been previously linked with gallstone disease (Serra *et al*, 1996) and cigarette smoking (Yagyu *et al*, 2008). It has been suggested that cyclooxygenase (COX) inhibition might have potential for chemoprevention in cholangiocarcinoma as COX-2 expression inhibits apoptosis in cholangiocarcinoma cells (Sirica *et al*, 2001). We set out to examine the role of the above risk factors in a large UK primary care-based population and, for the first time, to assess whether non-steroidal anti-inflammatory drugs (NSAIDs) protect against cholangiocarcinoma and gall bladder cancer.

## Materials and methods

The General Practice Research Database (GPRD) is a large longitudinal primary care database containing the primary care records of more than 8 million patients in the UK. The GPRD data are audited to ensure that at least 95% of medical events and prescriptions are satisfactorily recorded (Walley and Mantgani, 1997) and have been shown to provide results consistent with other UK data sources (Hollowell, 1997).

We identified people in the GPRD between 1987 and March 2002 with a recorded diagnosis of cholangiocarcinoma or gall bladder cancer using the Oxford Medical Information System and Read Clinical Classification codes. Cases were categorised into three groups: definite cholangiocarcinoma, definite gall bladder cancer, and a group containing patients who had both types of cancer coded or had codes that could not distinguish between them. The index date for cases was taken as the date of first diagnosis of biliary cancer. A total of 10 controls were matched to each case by GP practice, sex and age (within 5 years of the case). We excluded controls with less than 1 year of exposure data prior to case diagnosis.

Risk factors of interest were history of liver disease (including cirrhosis, hepatitis, primary sclerosing cholangitis, gallstone-related disease) or diabetes, prior NSAID use and lifestyle factors (smoking, alcohol and body mass index (BMI)). Any diagnoses occurring later than 6 months prior to the index date were excluded. Non-steroidal anti-inflammatory drug use was defined as any prescription of a drug listed under section 10.1.1 of the *British National Formulary* (No. 54). Analyses were repeated with exposure being classified by use at different time points preceding case diagnosis. We also repeated the analysis by excluding prescriptions for aspirin to ascertain the effect of non-aspirin NSAID use. Smoking status and alcohol intake were recorded at 1 year prior to the date of case diagnosis (or closest record prior to this). For alcohol intake, ‘problem drinkers’ were defined as those with a GPRD code indicating alcohol misuse at any time prior to diagnosis. Body mass index was calculated as weight in kg/height in m^2^. A separate category was created for the smoking, alcohol or BMI variables related to participants who had missing data.

Data were analysed using conditional logistic regression, with results presented as odds ratios (ORs) with 95% confidence intervals (CIs). Results were adjusted *a priori* for smoking, alcohol and BMI, with NSAID use also adjusted for gallstone disease diagnosis. Analyses were performed using the entire case group, and then separately for cholangiocarcinoma and gall bladder cancer cases. Stata version 10.0 was used for all analyses.

## Results

### Selection of cases and controls

We identified 611 cases, 372 cholangiocarcinomas, 184 gall bladder cancers and 55 unspecified biliary cancers. In total, 5760 controls were matched to the cases. The median duration of exposure data available prior to the date of case diagnosis was 4.8 years for cases (range 1.0–12.4 years) and 4.4 years for controls (range 1.0–12.5 years). The percentage of male cases was greater for cholangiocarcinoma (48.4%) than for gall bladder cancer (26.1%), whilst the mean age at diagnosis was similar for the two cancer types (71.3 (s.d. 12.0) years *vs* 72.0 (s.d. 11.1) years).

### Chronic liver disease and diabetes

Only small numbers of viral hepatitis (*n*=3), cirrhosis (*n*=2) and PSC (*n*=1) occurred among cholangiocarcinoma cases and none occurred among cases of gall bladder cancer. Only gallstone disease occurred with sufficient frequency to allow appropriate comparison of cases and controls, which was associated with a more than three-fold risk of gall bladder cancer and almost two-fold risk of cholangiocarcinoma (Table 1). The prevalence of diabetes was 8.5% in cases, which was significantly higher than the 5.9% in controls (OR=1.39; 95% CI, 1.01–1.90).

### NSAID use

A previous NSAID prescription was received by approximately 50% of both cases and controls, with no significant difference between the groups overall (OR=0.95; 95% CI, 0.80–1.13). In the interval 24–36 months preceding case diagnosis, there was significantly more NSAID use among cholangiocarcinoma cases (OR=1.34); however, in all other time intervals and among gall bladder cases ORs were close to 1 (Table 1). When we repeated this analysis for non-aspirin NSAIDs the findings did not change materially, with a slightly increased risk for cholangiocarcinoma in the 24–36-month period preceding diagnosis (OR=1.32; 95% CI, 1.00–1.75) and ORs closer to 1 in all other instances (data not shown).

### BMI, cigarette smoking and alcohol

We found that obese patients (BMI⩾30 kg/m^2^) had 1.5 times the risk of both cholangiocarcinoma and gall bladder cancer when compared with those with BMI <25 kg/m^2^ (Table 1). An increased risk was also observed among cigarette smokers, which was significant both among the entire series of cases (OR=1.48) and for the individual cancer types. We found neither current alcohol use nor problem alcohol drinking to be associated with either cancer (Table 1).

## Discussion

This is the first epidemiological study to examine the potential for chemoprevention of cholangiocarcinoma by the use of NSAIDs. Cyclooxygenase-2 is overexpressed in cholangiocarcinoma cells (Wu *et al*, 2002), is involved in apoptosis in these cells (Sirica *et al*, 2001), and its inhibition by Celecoxib has been shown *in vivo* to induce apoptosis in them (Wu *et al*, 2003). Despite this, no benefit from NSAIDS was found among our study group. Protective effects of NSAIDs on other GI tumors have been shown earlier using the same data set and methods similar to those we have used (Langman *et al*, 2000). A *post hoc* power calculation showed that our study had 90% power to detect a 25% reduction in risk with previous NSAID use; hence, our negative result was not because of lack of statistical power. One weakness of our study with respect to NSAIDs is that owing to their limited use in the UK there were not enough data to permit us to examine COX-2 inhibitors separately from other NSAIDs. This does not invalidate our results for NSAIDs, but leaves open the possibility that a more selective COX-2 inhibition may have a chemoprotective effect.

Our study confirms a number of earlier-described associations with other risk factors. We have been able to not only confirm the long-recognised association of gallstone disease (OR 2.34), but also reproduce significant associations with smoking (OR 1.48) and obesity (OR 1.58 for comparison of BMI<25 and BMI⩾30). We also found an association with diabetes of a magnitude similar to that reported in a recent publication from the SEER study (Welzel *et al*, 2007). We did not, however, in contrast to previous research (Shaib *et al*, 2007), find an association with alcohol intake. Exposure to PSC, cirrhosis or viral hepatitis was very rare within our study, hence greatly limiting our power to examine these factors.

Our results are not susceptible to selection bias as our study is population-based, using all available cases and appropriately selected controls from the same population. Nor, given the prospective recording of exposure data, is it prone to ascertainment bias beyond that because of the increased medical attention to which cases will inevitably be exposed in the run-up to and after their diagnosis. We have attempted to minimise this bias that might affect some of our risk estimates (such as those with diabetes and PSC) by excluding exposures later than 6 months before diagnosis from our analysis. One limitation of our study is that unlike in earlier studies, we are unable to say whether cholangiocarcinoma is intra- or extra-hepatic, nor to access histological or radiological records to validate the diagnosis. We think it unlikely that UK general practitioners would make a diagnosis of cholangiocarcinoma, and so we believe that the diagnoses are as secure as those in UK secondary care. Much of the quoted literature, however, refers specifically to intra-hepatic cholangiocarcinoma; therefore, if the risk factors for carcinogenesis vary by site within the biliary epithelium, this might explain why viral hepatitis and cirrhosis (if they affect intrahepatic more than extrahepatic ducts) occur less frequently in our study when compared with others (Donato *et al*, 2001).

In summary, this large population-based case–control study of biliary carcinomas has shown no protective effect from NSAIDs. It has, though, confirmed the increased risk of diabetes mellitus and gallstone disease, which earlier studies have found for cholangiocarcinoma, and has provided population-based estimates of the magnitudes of these associations. It has also highlighted the role of PSC, viral hepatitis and cirrhosis, but shows that although cholangiocarcinoma risk may be high in people with these risk factors, they explain little of the disease occurrence.

## Figures and Tables

**Table 1:** Risk factors for cholangiocarcinoma and gall bladder cancer: analysis with adjustment for cigarette smoking, alcohol consumption and BMI^a^

	**Controls**	**Cases**					
	**(*n*=5760)**	**All (*n*=611)**		**Cholangiocarcinoma (*n*=372)**		**Gall bladder cancer (*n*=184)**	
	***N* (%)**	***N* (%)**	**OR (95% CI)**	***N* (%)**	**OR (95% CI)**	***N* (%)**	**OR (95% CI)**
*History of diabetes or liver disease* >*6 months prior to case diagnosis*							
Diabetes mellitus	342 (5.9)	52 (8.5)	1.39 (1.01–1.90)	35 (9.4)	1.48 (1.00–2.17)	16 (8.7)	1.43 (0.81–2.52)
Cirrhosis	6 (0.1)	2 (0.3)	Not estimable	2 (0.5)	Not estimable	0 (0.0)	Not estimable
Viral hepatitis (excluding type A)	23 (0.4)	3 (0.5)	Not estimable	3 (0.8)	Not estimable	0 (0.0)	Not estimable
Primary sclerosing cholangitis	1 (<0.1)	1 (0.2)	Not estimable	1 (0.3)	Not estimable	0 (0.0)	Not estimable
Gallstone disease	268 (4.7)	66 (10.8)	2.34 (1.75–3.14)	33 (8.9)	1.78 (1.19–2.67)	27 (14.7)	3.57 (2.19–5.81)
							
*Any NSAID prescription (stratified by interval preceding case diagnosis)* b							
>6 months	3004 (52.2)	321 (52.5)	0.95 (0.80–1.13)	209 (56.2)	1.00 (0.80–1.26)	88 (47.8)	0.87 (0.63–1.21)
6–12 months	1419 (24.6)	159 (26.0)	1.04 (0.86–1.27)	108 (29.0)	1.12 (0.88–1.42)	40 (21.7)	0.90 (0.61–1.33)
12–24 months	1467 (30.9)	167 (32.4)	1.01 (0.82–1.24)	109 (34.3)	1.05 (0.81–1.35)	46 (29.1)	0.90 (0.61–1.32)
24–36 months	1188 (29.9)	152 (34.6)	1.19 (0.96–1.48)	108 (38.3)	1.34 (1.03–1.74)	39 (31.0)	1.03 (0.67–1.58)
36–60 months	909 (35.3)	114 (39.2)	1.17 (0.90–1.53)	77 (39.5)	1.21 (0.88–1.68)	32 (40.0)	1.09 (0.65–1.84)
							
Current cigarette smoker^c^	759 (20.9)	108 (26.8)	1.48 (1.16–1.89)	70 (27.5)	1.38 (1.01–1.87)	28 (24.8)	1.61 (1.00–2.62)
							
*BMI index* (*kg*/*m*^*2*^)^c^							
<25	1380 (45.6)	132 (39.1)	1.00	88 (39.1)	1.00	36 (41.9)	1.00
25–<30	1156 (38.4)	137 (40.5)	1.28 (0.99–1.66)	93 (41.3)	1.33 (0.97–1.82)	31 (36.1)	1.03 (0.62–1.72)
⩾30	471 (15.7)	69 (20.4)	1.58 (1.15–2.16)	44 (19.6)	1.52 (1.03–2.24)	19 (22.1)	1.51 (0.83–2.75)
							
*Alcohol consumption* c							
Alcohol abstainer	678 (23.1)	81 (24.4)	1.00	50 (23.5)	1.00	26 (28.6)	1.00
Alcohol user	2237 (76.2)	249 (75.0)	0.93 (0.70–1.23)	162 (76.1)	0.90 (0.63–1.29)	65 (71.4)	0.99 (0.59–1.64)
Problem drinker	19 (0.7)	2 (0.6)	Not estimable	1 (0.3)	Not estimable	0 (0.0)	Not estimable

BMI=body mass index; CI=confidence interval; NSAID=non-steroidal anti-inflammatory drug; OR=odds ratio.

a Smoking was categorised as current smoking, non-smoking, missing; alcohol consumption as non-drinking, current drinking, problem drinking, missing; and BMI into four categories, including missing.

b NSAID use is also adjusted for history of gallstone disease in the previous 6 months.

c Smoking data were unavailable for 34% of cases and 39% of controls, alcohol data for 46% of cases and 49% of controls, and BMI for 45% of cases and 48% of controls. Percentages and ORs for these variables were based on cases and controls with available data.

All ORs were obtained using conditional logistic regression for matched cases and controls. ORs were not presented when either the number of exposed cases or the number of exposed controls was <5 owing to unreliability of the accompanying 95% CI in these instances.
